# Protocol for applying expansion microscopy to the study of mammalian neuromuscular junctions

**DOI:** 10.1016/j.xpro.2025.104272

**Published:** 2025-12-12

**Authors:** Abdullah Ramadan, Thomas M.D. Sheard, Abrar Alhindi, Philippa A. Rust, Ross A. Jones, Thomas H. Gillingwater, Izzy Jayasinghe

**Affiliations:** 1 Edinburgh Medical School: Department of Anatomy, University of Edinburgh, Edinburgh, UK; 2 College of Sciences and Health Professions, King Saud bin Abdulaziz for Health Sciences, Jeddah, Saudi Arabia; 3 School of Biosciences, Faculty of Science, University of Sheffield, Sheffield S10 2TN, UK; 4 Clinical Anatomy Department, Faculty of Medicine, King Abdulaziz University, Jeddah, Saudi Arabia; 5 Hooper Hand Unit, St John’s Hospital, Howden Road West, Livingston, UK; 6 Euan MacDonald Centre for Motor Neuron Disease Research, University of Edinburgh, Edinburgh, UK; 7 EMBL Australia Node in Single Molecule Science, Department of Molecular Medicine, School of Medical Sciences, UNSW Sydney, Sydney, NSW, Australia

**Keywords:** microscopy, model organisms, neuroscience

## Abstract

Expansion microscopy (ExM) is a tissue-swelling technique that enables super-resolution imaging through a specialized preparation process that physically expands stained biomolecules within a fixed sample. Here, we detail a protocol to apply the 4× ExM technique to neuromuscular junctions (NMJs) from both human and mouse muscle preparations. We describe steps for muscle fixation, microdissection, staining, gelation, and digestion. We then detail procedures for expansion, mounting, imaging, analysis, and quantification. This protocol can be used to reveal nanoscale anatomical NMJ features.

For complete details on the use and execution of this protocol, please refer to Ramadan et al.^1^

## Highlights

• Expansion microscopy of mammalian muscle: dissection, fixation, and NMJ labeling
• Instructions for gel embedding, digestion, and gel expansion for thick muscular samples
• Guidance on imaging and analyzing expanded NMJs at super-resolution scale

## Abstract (editor highlights)

Publisher’s note: Undertaking any experimental protocol requires adherence to local institutional guidelines for laboratory safety and ethics.

## Graphical abstract

**Figure:**
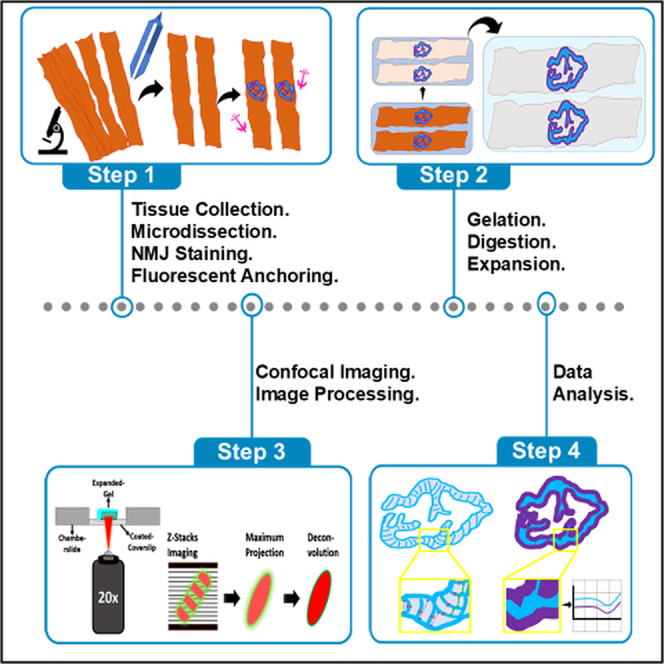

## Before you begin

Expansion microscopy (ExM) was first introduced over a decade ago.^2^ Since then, numerous adapted protocols have emerged, facilitating high-resolution imaging of a range of different biological tissues and research samples.^3^ The concept of super-resolution (SR) ExM imaging is achieved through a specialized sample preparation process, which results in the physical expansion of stained biomolecules.^4^ Here, we present a detailed protocol for applying the classical 4× ExM protocol to investigate synaptic components of the neuromuscular junction (NMJ) in order to reveal its nanoscale morphological features.^1^ We demonstrate the feasibility of our protocol in thick muscular specimens from both human biopsy samples and mouse tissues, using dorsal interosseous (DIO) muscles from the hand and forepaw respectively. This protocol is relatively straightforward and convenient, taking five subsequent days for completion and requiring only standard laboratory equipment. The workflow is characterized by applying a robust, previously-optimized NMJ immunohistochemical staining protocol on muscle samples^5^ (with particular emphasis on microdissection) and adopting a classical 4x ExM protocol that was developed to study cardiac ryanodine receptor clusters in cardiomyocytes in vitro,^6^ but with modifications required in order to make it applicable when working with thick skeletal muscle samples. The protocol also incorporates a simplified method for analyzing NMJ components using the open-source software Fiji. Finally, we highlight key challenges and limitations, along with proposed potential troubleshooting strategies. Our main purpose is to make this protocol widely available to the NMJ research community, as it offers a powerful but practical approach for visualizing NMJ nanostructure in a way that doesn’t require the more extensive resources and technology needed for SR imaging techniques.^3^

For our initial study^1^ the decision to use the mouse DIO muscle from the forelimb paw was driven by the aim to identify an equivalent muscle to that obtained from human surgical biopsies, enabling comparative analysis between the two species using the ExM technique. However, the protocol is widely applicable to any skeletal muscle. In our experience, particularly for human muscle surgical biopsy samples, the key determinant of success is keeping the time between sample removal and sample fixation as short as possible. Our findings are based on prompt tissue collection from recently harvested surgical muscle biopsies (∼5–10 minutes between removal and fixation) or freshly dissected mice (∼10–5 minutes between culling and muscle fixation).

In order to fluorescently label acetylcholine receptors (AChRs) at the NMJ, conjugates comprised of Alpha-bungarotoxin (α-BTX) with a fluorescent dye are routinely used.^7^^,^^8^^,^^9^^,^^10^ Since there are different conjugates, we found that the α-BTX-Alexa Fluor 488 conjugate is the best choice compared to tetramethyl rhodamine (TRITC) α-BTX. This is an important note since not all fluorescent labels exhibit the same level of retention in Expansion Microscopy (ExM), with some demonstrating a significantly lower percentage of fluorescence retention.^11^ Also, in our protocol, we prioritize the labeling of AChRs before applying primary antibodies targeting other synaptic proteins, making it possible to confirm the presence of the endplate in human muscle biopsy samples.^1^ However, the sequence of AChR and antibody labeling is not critical to the overall process and can be adjusted as needed.

### Innovation

This protocol introduces several key innovations that enable the ExM application to whole-mount human and mouse muscle biopsy samples for detailed NMJ visualization. Traditional ExM workflows were optimized for thin tissue sections or cell cultures, in which gelation occurs on flat slides with minimal concern for sample movement. In contrast, the handling of free-floating muscle fibers presents unique challenges, including sample displacement during gel embedding, distortion of NMJ orientation, and non-uniform polymerization leading to gel cracking and distortion. To overcome these limitations, we developed a modified mounting strategy by following these steps.

1. Transferring the dissected muscle biopsy samples onto poly-L-lysine–coated coverslips before gelation.
2. Flipping the dissected muscle fibers into gel solution. This stabilized the tissue architecture, preserved fiber orientation, and ensured homogeneous gel formation.
3. Incubate the gels for digestion in 37°C for 18–20 h. This will optimize the digestion process efficiently (clearing the tissues and maintaining fluorescence integrity).

The above-mentioned adjustments substantially improve the isotropic expansion, avoid gel distortions, and yield consistent expansion factors (∼3.5x) across both murine and human samples. Hence, transform ExM into a robust, reproducible, and high-resolution approach for studying NMJ morphology within intact muscle biopsies, offering a practical and cost-effective alternative to an advanced SR microscopy platform. By markedly lowering the technical and financial barriers traditionally associated with nanoscale NMJ imaging, this protocol makes SR structural analysis accessible to any laboratory equipped with a standard confocal microscope.

### Institutional permissions (if applicable)

Human muscle tissue was obtained with approval from the NHS Grampian Research Ethics Committee (REC ref. 20/NS/0008; Protocol number: AC18077; IRAS project ID: 244717). All procedures involving animals were carried out under a UK Home Office Project Licence (PPL PP1567597) in accordance with institutional and national regulations.

### Ethics statement

All human muscle biopsy procedures were conducted in accordance with the ethical standards of the NHS Grampian Ethics Committee and the principles outlined in the Declaration of Helsinki. Written informed consent was obtained from all donors prior to tissue collection (REC ref. 20/NS/0008; Protocol number: AC18077; IRAS project ID: 244717). All animal experiments were approved by the UK Home Office under Project Licence PPL PP1567597 and performed in accordance with the Animals (Scientific Procedures) Act 1986 and institutional ethical guidelines.

### Preparation steps

#### Muscle sample collection and fixation

**Timing: ∼3 h**

4. Coordinate with the clinical team (for human surgical biopsies) to ensure rapid transfer of the excised muscle to 4% paraformaldehyde (PFA) pre-dissolved in 1× PBS.
5. Place the muscle sample in 4% PFA (Electron Microscopy Sciences, Cat#15710) for 2 hours, within 5–10 minutes post-excision for human samples or within 10–15 minutes post-culling for mouse tissue.
6. Use a screw-cap specimen vial for immersion fixation at room temperature, which is around 20°C–27°C (see Step-by-step Method for detailed fixation procedure).

**CRITICAL:** Prompt fixation is essential to preserve synaptic integrity.

#### Muscle dissection and fiber teasing

**Timing: ∼30–60 min per sample**

7. Transfer fixed muscle samples to a Sylgard-coated Petri dish placed under a stereomicroscope. Use fine-tipped forceps to tease the muscle into bundles of approximately 5–10 fibers. Use curved forceps to stabilize the sample and use straight forceps to gently separate muscle fibers.
8. Carefully remove visible connective tissue from around and between the fibers. Ensure fibers remain intact and undamaged to facilitate uniform expansion and antibody penetration.

***Note:*** For a detailed dissection workflow, please refer to Figure 2 in the full protocol below.

#### Gel chamber allocation and coverslip coating

**Timing: ∼3 h (including drying time; prepare 1 day before gelation)**

9. Cut square #1.5 coverslips (22 × 22 mm) for mounting the muscle samples.
10. Coat each coverslip with 200 μL of poly-L-lysine solution (Sigma-Aldrich, P8920-100mL).
11. Incubate coated coverslips at room temperature (RT = 20°C–27°C) for 30 minutes.
12. Rinse gently with sterile dH_2_O and allow to air-dry completely in a sterile environment.
13. Allocate one coverslip per muscle bundle to ensure one sample per gel chamber.

## Key resources table

**Table:** 

REAGENT or RESOURCE	SOURCE	IDENTIFIER
**Antibodies**		

Mouse anti-SV2 IgG antibody (1:50 dilution)	Developmental Studies Hybridoma Bank	Cat#2315387; RRID: AB-2315387
Mouse anti-NaV1.4 N255/38 antibody (1:500 dilution)	Antibodies Incorporated	Cat# N255/38; RRID: AB-2877201
Alexa 594-goat anti-mouse IgG (1:400 dilution)	Thermo Fisher Scientific	Cat# A-11005; RRID: AB-2534073
Alexa 488-α-bungarotoxin (1:500 dilution)	Thermo Fisher Scientific	Cat# B13422

**Biological samples**		

Human dorsal interosseous muscle biopsies	This paper	N/A
FVB mice (12 weeks old, both sexes)	Biological Research Resources, University of Edinburgh	N/A
Human dorsal interosseous muscle biopsies	This study	N/A

**Chemicals, peptides, and recombinant proteins**		

Paraformaldehyde (PFA)	Electron Microscopy Sciences	Cat#15710
Bovine Serum Albumin (BSA)	Sigma-Aldrich, UK	Cat#A4503
Sodium Acrylate (SA)	Sigma-Aldrich, UK	Cat#408220-25g
Acrylamide	Sigma-Aldrich, UK	Cat#A9099-25g
N,N-methylenebisacrylamide	Sigma-Aldrich, UK	Cat#274135
Sodium Chloride (NaCl)	Sigma-Aldrich, UK	Cat#S7653-1kg
N,N,N′,N′-Tetramethylethylenediamine (TEMED)	Sigma-Aldrich, UK	Cat#T7024
Ammonium persulfate (APS)	Thermo Fisher Scientific, UK	Cat#A3678-25g
Tris	Sigma-Aldrich, UK	Cat#AM9855g
Ethylenediaminetetraacetic acid (EDTA)	Sigma-Aldrich, UK	Cat#EDS-100g
Guanidine Hydrochloride	Sigma-Aldrich, UK	Cat#G327225g
Triton X-100	Sigma-Aldrich, UK	Cat#T9284
Acryloyl-X (AcX)	Thermo Fisher Scientific, UK	Cat#A20770, 5mg
DMSO	Biotium, USA	Cat#90082-BT
Poly-L-lysine	Sigma-Aldrich, UK	Cat#P8920-100mL
PBS (10X), pH 7.4	Thermo Fisher Scientific	Cat#70011044
Proteinase K	New England Biolabs, UK	Cat#P8107S, 2 mL
Fluorescent microspheres (0.1 μm diameter)	Thermo Fisher Scientific, USA	Cat#T7279

**Software and algorithms**		

Huygens Software: Classic Maximum Likelihood Estimation (CMLE)	Scientific Volume Imaging (SVI)	https://svi.nl/Huygens-Confocal-Software
Fiji	ImageJ	https://imagej.net/imagej-wiki-static/Citing
GraphPad Prism	GraphPad Software, USA	Version 10

**Other**		

Acrylic slide chambers	Ponoko	https://www.ponoko.com/
Stereomicroscope	Nikon	SMZ1270
Diamond-tipped glass cutter	Generic/electrical stores	N/A

## Materials and equipment

***Note:*** Final volume adjusted to 9.4 mL.

**CRITICAL:** Sodium acrylate is classified as an environmental hazard. Handle with gloves and appropriate protective equipment in a fume hood.

**Table 1:** Chemicals and their concentrations used to prepare the monomer solution

Reagent	Stock concentration	Volume to add
Sodium acrylate	38 g/100 mL (3.8 g/10 mL)	2.25 mL
Acrylamide	50 g/100 mL (5 g/10 mL)	0.5 mL
N,N′-methylenebisacrylamide	2 g/100 mL (0.2 g/10 mL)	0.75 mL
NaCl	29.2 g/100 mL (2.92 g/10 mL)	4 mL
10x PBS	–	1 mL
dH_2_O	–	0.9 mL

Combine reagents sequentially in a sterile 15 mL conical tube and mix gently by inversion. Aliquot into ≈300 μL portions in microtubes to avoid repeated freeze–thaw cycles. Store at −20°C for up to 2 months.

***Note:*** Proteinase K: Add freshly before use at 100 μL per 10 mL digestive buffer.

**Table 2:** Chemicals and their concentrations used to prepare the digestive buffer

Reagent	Stock concentration	Volume to add
Tris (pH 8.0)	1 M	5 mL
EDTA	0.5 M	0.2 mL (to 1 mM final)
Guanidine Hydrochloride	Powder	7.642 g
Triton X-100	100%	0.5 mL
dH_2_O	–	Up to 100 mL

Add reagents sequentially to a sterile beaker and mix thoroughly until all solids dissolve. Aliquot into 10 mL portions (≈1 experiment each). Store at −20°C until required.

### Anchoring reagent (acryloyl-X working solution)

Anchoring is achieved by adding 0.1 mg/mL acryloyl-X (AcX; Sigma-Aldrich, UK) from an AcX stock of 10 mg/mL in DMSO (Biotium, USA) overnight (12–20 hours) at 4°C, which can be aliquoted and stored at −20°C for up to two months. On use, dilute at 1:100 in 1x PBS.

**CRITICAL:** AcX is light-sensitive. Handle in low-light conditions and store protected from light.

### Gelation mixture (300 μL per experiment)

Prepare the gelation solution immediately prior to use. In a sterile microcentrifuge tube, combine 282 μL of pre-prepared monomer solution (Table 1) with 6 μL of 1× PBS. Just before application, add 6 μL of 10% ammonium persulfate (APS), followed by 6 μL of 10% N, N, N′, N′-tetramethylethylenediamine (TEMED). Mix gently by pipetting, avoiding air bubbles, and use immediately to prevent premature polymerization.

**CRITICAL:** APS is an oxidizing agent, and TEMED is a skin and respiratory irritant. Handle both reagents in a fume hood while wearing gloves and safety goggles.

### Gel and imaging chamber preparation

To prepare the gel chamber, cover a standard glass microscope slide with a flat, even layer of Parafilm to serve as the chamber base. Use a diamond-tipped glass cutter to cut #1.5 square coverslips (22 × 22 mm) into rectangular pieces that serve as spacers (typically four per coverslip). Attach one spacer at each end of the Parafilm-covered slide using 2–3 μL of dH_2_O to enhance adhesion.

**Figure 1:**
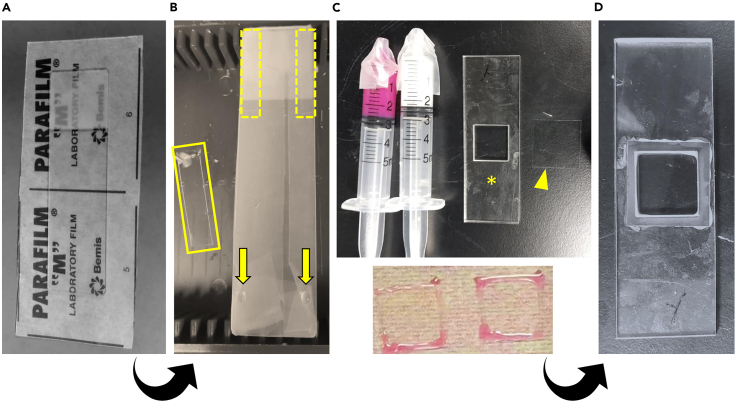
Preparation of gel and imaging chambers (A) A standard microscope slide is wrapped with Parafilm to serve as the base of the gel chamber, where polymerization occurs. (B) Small fragments of coverslip glass are used as spacers; one such piece is highlighted by a yellow box, while dashed boxes indicate the approximate positions where spacers are affixed. Yellow arrows denote the application of a droplet of water, which facilitates adhesion of the spacers to the slide. (C) A coverslip pre-coated with poly-L-lysine (yellow arrowhead) is affixed to the base using PinkySil silicone molding rubber. The silicone adhesive is prepared by mixing the pink and white components in a 1:1 ratio and applied along the edges of the coverslip to ensure secure attachment. (D) A custom acrylic chamber (yellow star) is subsequently positioned on top of the coverslip, and gentle pressure is applied to ensure complete and uniform adhesion to the underlying surface.

Separately, coat additional #1.5 coverslips (22 × 22 mm) with 200 μL of Poly-L-lysine solution (Sigma-Aldrich, P8920-100mL) per coverslip. Incubate at room temperature (20°C–27°C) for 30 minutes, rinse with sterile dH_2_O, and air dry completely. These will serve as the gel chamber roof, onto which the muscle samples are mounted (Figure 1).

For imaging chambers, use custom-fabricated acrylic chambers (e.g., RazorLab or Ponoko Laser Cutting). Square cutouts (18 mm) or circular cutouts (17 mm diameter) are compatible with standard coverslips. To affix the coverslip to the chamber base, apply PinkySil silicone moulding rubber (SR-PINKY-T, Barnes, AU), (Figures 1C and 1D) along the edges and press the acrylic body gently but firmly into place.

***Note:*** All coverslips should be cleaned and dried before assembly to ensure adhesion.

### Preparation of fluorescent microspheres for PSF estimation

To estimate the point spread function (PSF) under conditions mimicking Expansion Microscopy, 0.1 μm fluorescent microspheres were used as sub-diffraction point sources. A 1:1,000 working dilution was first prepared by vortexing 1 μL of bead stock in 999 μl of PBS. From this, 5-10 μL was added to 200 μl of PBS in an imaging chamber. After gentle mixing, z-stack images were acquired using a 20x air objective under the same imaging parameters as the expanded samples. Fields containing isolated, well-distributed beads (<10 per field of view) were selected for PSF extraction using Huygens software (which calculates the PSF automatically), with the appropriate channel chosen according to the emission wavelength of the beads.

***Note:*** It is advisable to perform the PSF calibration using microspheres (beads) at the start of a study or whenever imaging conditions are altered (such as switching objective lenses or the microscope). For routine experiments conducted under unchanged settings, repeating PSF calibration before each acquisition is not required.

## Step-by-step method details

### Muscle fixation and microdissection

**Timing: Day 1**

This section details the fixation and microdissection of muscle samples to preserve neuromuscular architecture and prepare tissues for subsequent labeling and expansion. Rapid fixation stabilizes structural integrity, while fine teasing of fibers enhances antibody penetration and promotes uniform gel expansion eventually.

**Figure 2:**
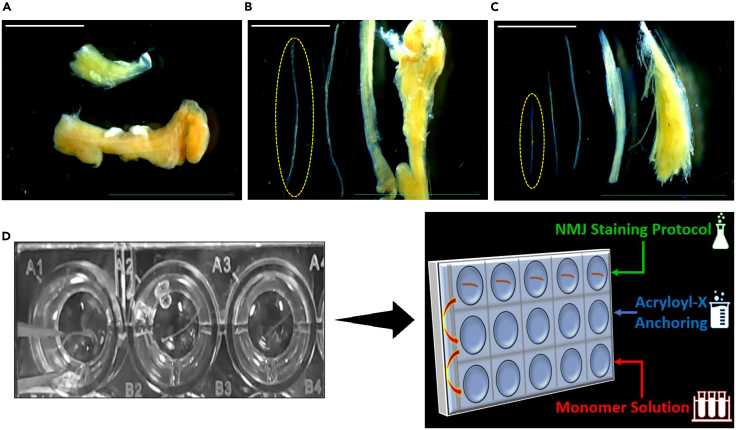
Muscle fiber preparation for ExM (A) Representative samples of dissected muscle tissue: mouse (top) and human (bottom). Both muscle types were freshly isolated and fixed in 4% PFA to preserve morphological integrity for whole-mount NMJ immunolabeling and subsequent ExM processing. (B) Microdissection of a human muscle sample. (C) The same teasing technique applied to mouse muscle samples. Individual fibers or small bundles were gently separated under a stereomicroscope using fine forceps to facilitate reagent penetration during staining and expansion. The yellow oval delineates the optimal size of teased fibers, ensuring sufficient tissue thinness for ExM protocol with preservation of native NMJ architecture. Scale bars represent 0.5 mm in (A, B and C). (D) Teased muscle fibers were transferred into individual wells of a 24-well plate, where the remaining steps of the ExM protocol were performed. This included immunostaining for NMJs, treatment with the anchoring agent (e.g., acryloyl-X, SE), and infiltration with monomer solution prior to gel polymerization and enzymatic digestion.

1. Sample Fixation:
  a. Immerse the muscle samples in 4% PFA (Aqueous Solution Electron Microscopy Sciences, 15710) in 1x phosphate-buffered saline (PBS) buffer for 1 hour at room temperature (20°C–27°C) within screw-cap specimen vials.
  b. Wash the samples with 1x PBS for three rounds, each for 10 minutes, to remove any residual fixative and prepare the specimen for further processing.
2. Microdissection:
  Dissect the muscle fibers in a 90 mm sealed Sylgard-coated Petri dish (Figures 2A–2C), under a stereomicroscope for visual guidance.
  ***Note:*** Depending on sample thickness and level of detail required, dissections were carried out using variable zoom settings, typically between 0.63x and 8x magnification. Large muscle fascicles must be carefully teased into small groups (≈5–10 fibers each), where each group is has a size of ∼1.5 mm × 1.0 mm of muscular tissue as (Figures 2A–2C) indicates. This can be achieved by the following steps:
  a. Use fine scissors and fine-tipped forceps, meticulously remove all visible connective tissue from the muscle specimen, including both the outer fascia and any connective tissue located between individual muscle fibers.
  b. Subsequently, gently tease apart the muscle bundles using a combination of straight and curved fine forceps. Employ the curved forceps to stabilize the sample while delicately separating the fibers with the straight forceps.
  c. Store the teased fibers in 4°C in 1x PBS until resuming the protocol on the next day.
    **CRITICAL:** The key to success in meticulous microdissection is cleaning the connective tissue and teasing the fibers as much as possible. This will allow for optimal penetration of antibodies and complete digestion, which is required for subsequent successful expansion.
    ***Note:*** Each individual sample within a single gel will ultimately determine the number of muscle fibers and, consequently, the number of NMJs available for analysis. Reducing the sample size as much as possible can improve tissue digestion and facilitate more uniform gel expansion. However, smaller samples may also contain fewer NMJs, thereby limiting the number that can be imaged.

### Neuromuscular junction staining

**Timing: Day 2 and 3**

This section explains in detail the NMJ staining protocol that has been used in this protocol.

3. Permeabilization and Blocking:
  a. Following microdissection, transfer the teased muscle fibers to a 24-well plate (Figure 2D).
  b. Add 4% Triton X (v/v) at RT on a rocker to facilitate antibody penetration (for permeabilization). The volume is variable, where the muscle fibers should be totally immersed in the buffer (in 24 well plates usually 900 μl is enough per well).
  c. Add the blocking solution containing 4% BSA (w/v) and 2% Triton X (v/v) (Sigma-Aldrich, UK) in RT on rocker (in 24 well plates usually 900 μl is enough per well).
4. Antibody Incubation, labeling and Biomolecule Anchoring:
  a. AChR Labeling**: Use** Alexa-488 α-Bungarotoxin (α-BTX, Alexa Fluor 488 conjugate, B13422, Invitrogen) to label the AChRs. The applied diluted ratio is (1:500), and the incubation time is 30 minutes at room temperature (20°C–27°C).
  b. Incubate the muscle bundles overnight (12–20 hours) at 4°C with one of the following primary antibodies:
    i. Mouse anti-SV2 IgG (Developmental Studies Hybridoma Bank, University of Iowa) at 1:50 dilution.
    ii. Mouse anti-NaV1.4 IgG (Anti-NaV1.4 Sodium Channel Antibody-N255/38, Antibodies Incorporated) at 1:500 dilution.
      ***Note:*** Primary antibodies were applied in a blocking solution containing 4% BSA and 2% Triton X.
  c. After primary antibody incubation, wash the samples four times with 1% PBS, for 20 minutes each.
  d. Incubate the samples with secondary Antibody: Alexa 594-Goat anti-mouse IgG (Thermo Fisher Scientific, Cat No: A-11005) was applied at 1:400 dilution (1x PBS) for 5 hours at RT.
  e. Samples were washed four times with 1% PBS, for 20 minutes each.
  f. At the end of day 3 (after finishing the staining protocol), wash the labeled muscle fibers two times with 1% PBS (10 minutes each). Then, within the same 24-well plates, add the anchoring regent by applying 0.1 mg/mL of anchoring reagent acryloyl-X (AcX; Sigma-Aldrich, UK) from an AcX stock of 10 mg/ml in (DMSO; Biotium, USA).
  g. Remove the 24-well plates immediately to dark storage at 4°C overnight (12–20 hours).
    ***Note:*** All of the aforementioned steps are applied in the same 24-well plate (Figure 2D the schematic). Transferring the muscle fibers within the wells by using forceps is safer and easier than trying to suction/add the regents by pipette (to avoid suctioning the fibers mistakenly). Also, the 24-well plate should be always wrapped with aluminum foil to prevent any potential photobleaching of the stained samples.
    ***Optional:*** We recommend the inclusion of a secondary-only control as an optional step, particularly when users introduce novel antibodies or staining combinations. This ensures that any non-specific binding of secondary antibodies is detected without significantly increasing experimental workload.

### Gelation and digestion (polymerization and homogenization)

**Timing: Day 4**

This section outlines the embedding of muscle fibers within a polymer matrix, followed by enzymatic digestion for the tissue to make it physically expandable.

5. Preparing the gel matrix (monomer solution application):
  a. In the same 24-well plates, wash the samples to remove any remaining anchoring solution with 1% PBS (900 μl per well) twice (10 minutes each) at RT on rocker. Then, add 300 μl of monomer solution per well. Table 1 explains the recipe of the monomer solution.
  b. Keep the 24-well plate at 4°C for 3 hours in dark (wrapped with aluminum foil).
  c. Place a standard glass microscope slide on top of a microscope slide storage box to serve as a stable working platform.
6. Preparation of gel chambers:
  a. Prepare the gel chamber as previously described in gel and Imaging Chambers Preparation (materials and equipment setup section), by wrapping the glass slide with semi-transparent Parafilm and adding coverslip spacers (Figure 1A).
  b. Position one spacer at each end of the Parafilm-covered glass slide to define the chamber height (Figure 1B).
    ***Note:*** To enhance adhesion between the spacers and the slide surface, apply 2–3 μL of dH_2_O at the interface between each spacer and the slide.
  c. Remove the 24-well plate from the refrigerator. In a dimly lit room, carefully transfer the muscle fibers from the wells onto individual coverslips (#1.5) that have been pre-coated with poly-L-lysine. Ensure that only one muscle sample is placed per coverslip (Figure 3A).

**Figure 3:**
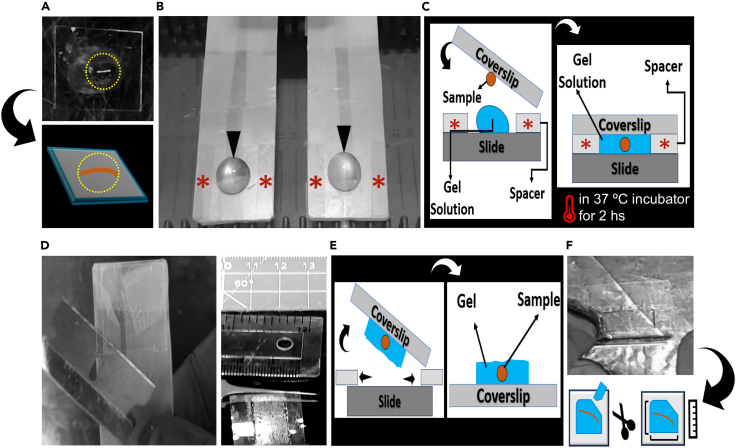
Embedding and gelation steps for ExM of muscle tissue (A) The teased muscle sample was carefully mounted onto a glass coverslip (18 × 18 mm, No. 1.5) previously coated with 250 μL of 0.1% poly-L-lysine solution for 1 hour to enhance tissue adhesion. The yellow dashed circle highlights the position of the muscle sample on the coverslip. (B) Gel polymerization (gelation) was initiated by sequential preparation of a 300 μL gelation mixture, as indicated by the black arrowheads. Of this mixture, 150 μL was applied into each gel chamber between two spacers (marked by red stars), yielding two individual expandable gels per chamber. (C) The tissue-mounted coverslip was gently inverted onto the gelation chamber, allowing contact between the muscle sample and the gel solution. Polymerization was carried out for 2 hours at 37°C in a humidified incubator. (D) Following gelation, the upper coverslip (to which the sample and gel adhered) was carefully separated from the chamber using a sterile blade. The spacers were simultaneously removed. (E) Schematic illustration of the gel detachment step, showing the muscle-bearing gel adhered to the upper coverslip. (F) The gel was trimmed with a sterile blade into an asymmetric shape to aid later orientation during imaging and analysis. Pre-expansion gel dimensions were recorded at this stage to facilitate accurate calculation of the expansion factor.
  d. Prepare the gel solution in a 5 mL Eppendorf tube. Use 282 μl of monomer solution (as Table 1 explains), 6 μl PBS, 6 μl 10% of Ammonium persulfate APS, and 6 μl 10% of N,N,N0,N0 Tetramethylethylenediamine (TEMED).
  e. Keep it on ice or in a chilled environment to prevent premature polymerization (details in the materials and equipment **setup** section).
  f. Carefully invert the coated coverslip—bearing the attached muscle fibers—onto the Parafilm-wrapped microscope slide, such that the coverslip forms the roof of the gel chamber, while the Parafilm-wrapped slide serves as the chamber floor.
    ***Note:*** Now the gel solution, along with the muscle sample, will be enclosed between these two surfaces (the slide and the coverslip, bottom and top, respectively). The spacers positioned at either end of the chamber provide additional height, creating sufficient space for gel polymerization to occur uniformly between the roof and floor.
  g. Close the box and transfer it into a 37°C incubator for 2 hrs., where the gelation solution will polymerize. Figure 3C presents a schematic overview illustrating the procedures detailed in this step.
    **CRITICAL:** Gel chambers should be prepared immediately prior to use. We do not recommend batch-preparation and storage, as this may compromise chamber integrity and gel polymerization quality. Hence, effect negatively on the expansion and imaging eventually.
    ***Note:*** After transferring the muscle fiber to the coverslip, proceed immediately (no waiting period is required for fiber attachment).
  h. Remove the microscope slide box containing the polymerized gels from the 37°C incubator.
  i. Allow it to cool at room temperature (20°C–27°C) for approximately 2 minutes.
  j. Carefully extract the gelation chamber and place the box aside.
  k. Gently insert a razor blade beneath the spacers from the side of the chamber to initiate separation. Then, carefully lift and invert the coverslip such that the gel is now facing upwards, with the coverslip positioned underneath.
    ***Note:*** The spacers may either remain adhered to the slide or detach during this process. Then, cut the gel into an asymmetrical shape to easily determine the orientation (Figures 3D–3F).
  l. At this stage, record the pre-ExM measurement as shown in (Figure 3F).
    ***Note:*** Using a clean ruler or calliper, measure and record the physical dimensions (X and Y axes) of the gel after polymerization and before enzymatic digestion to record the pre-ExM dimensions.
    **CRITICAL:** The expansion factor is later derived by comparing this pre-expansion size to the corresponding post-expansion dimensions. So, the expansion factor is mandatory for normalizing morphological measurements obtained after expansion to their original scale eventually.
7. Digestion:
  a. During the gelation period (which takes two hours), prepare the digestion solution by mixing proteinase K (ProK) with the pre-prepared digestion buffer (Table 2) in conical centrifuge tubes (10 ml) (as detailed in the materials and equipment setup section).
    **CRITICAL:** The mixture should be prepared at room temperature (20°C–27°C) using a volumetric ratio of 1:100. Specifically, add 100 μL of proteinase K to 10 mL of the digestion buffer.
    ***Note:*** Typically, the gel remains stuck to the underlying coverslip. Hence, use forceps to transfer the gel into 6-well plates where 2 ml of digested buffer can be added per well (per gel). (Figures 4A and 4B)
    **Figure 4:**
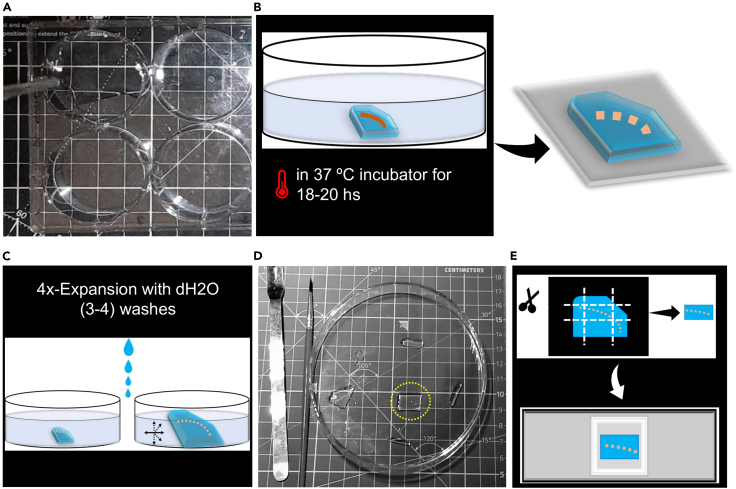
Muscle sample digestion and gel expansion (A) After polymerization, gels (adhered to coverslips) were transferred to a six-well plate (one gel per well) and fully immersed in digestion buffer containing Proteinase K (1:100 dilution; 2–3 mL per well). Digestion was carried out at 37°C for 18–20 hours to ensure uniform expansion and minimize artefacts. (B) Schematic illustrating the digestion step. (C) Gels were then transferred to a 150 mm Petri dish and washed four times (30 min each) in ddH_2_O in darkness. After detaching from the coverslip and reaching stable size, gel dimensions were recorded to confirm 3.5–4× expansion. (D) Expanded gels were trimmed, oriented, and mounted using coverslips, metal spatulas, and a paintbrush. (E) Finally, transfer the gel into the imaging chamber (the custom acrylic chambers fitted with #1.5 coated coverslips).
  b. Wrap the 6-well plate with aluminum foil and place it in a 37°C incubator for 18–20 hrs.
    **CRITICAL:** Improper digestion leads to distortion (with visible cracks) upon expansion. However, a longer period of digestion will damage the fluorophores and lead to dim imaging.

### Expansion, mounting, and imaging

**Timing: Day 5**

This section describes the physical expansion of the polymerized gel, preparation of imaging chambers, and acquisition of SR images of expanded NMJs. Sequential water exchanges drive isotropic gel swelling, while precise mounting and immediate imaging preserve hydration and structural fidelity, ensuring accurate morphometric analysis of NMJ architecture.

***Note:*** Ensure a dark environment during these stages to minimize the chances of photobleaching.

8. Gel Expansion:
  a. Remove the 6-well plate from the incubator and transfer gels into 150 mm petri dishes (one gel per dish).
  b. Fill the dishes with pure water dH_2_O for 45 mins. It is important to ensure total immersion of the gel (Figure 4C).
  c. Carefully remove the water surrounding the gel using an electronic pipette controller, which provides greater precision and faster time. Repeat the process of water removal and rehydration with fresh distilled water multiple times (typically three to four cycles, each one takes 40 minutes) until the gel reaches its maximum expansion (plateau phase), indicated by no further noticeable increase in size.
    ***Note:*** During this process, the expanded gel will gradually detach from the coverslip by default without the need for manual intervention.
  d. Re-measure the expanded gel to document the post-ExM dimensions (once the gel has been fully expanded), in order to determine the expansion factor.
9. Imaging Chamber Preparation:
  a. Coate Square glass coverslips (#1.5, 22 × 22 mm) with 200 μL of Poly-L-lysine solution (per coverslip) and incubate them at room temperature (20°C–27°C) for 30 minutes. After incubation, the coverslips are thoroughly rinsed with dH_2_O and allowed to air dry completely before use.
  b. Acrylic chambers (custom-made), which were laser-cut from 3 mm clear acrylic sheets and fabricated by Ponoko Ltd. A 22 × 22 mm No. 1.5 coated glass coverslip was affixed to the base of each chamber using Pinkysil silicone molding elastomer molding rubber (SR-PINKY-T, Barnes, AU), as described in the materials and equipment Setup section above. (Figures 1C and 1D).
10. Gel Mounting:
  a. Carefully trim the expanded gel using appropriate tools (cut it by using the edge of a coverslip and remove the trimmed parts by using a small paintbrush). (Figure 4D).
  b. Using a flat metal spatula and coverslip, transfer the prepared gels into the customized acrylic imaging chamber (Figure 4E).
    **CRITICAL:** The digested muscle fibers are not visible via bright-field illumination or the naked eye; if fibers are discernible within the gel, this likely indicates incomplete digestion which can lead to distortion and sub-optimal expansion factor. It is instructive to work based on prior knowledge or notes on the location of the NMJs (from fluorescent labeling) and to trim with the maximum margin allowable between the expected boundary of the specimen and the new boundary of the gel (as illustrated in Figure S1).
  c. Transfer the imaging chamber (loaded with gels) into a microscope slide box to ensure a dark environment (Figure 5A).

**Figure 5:**
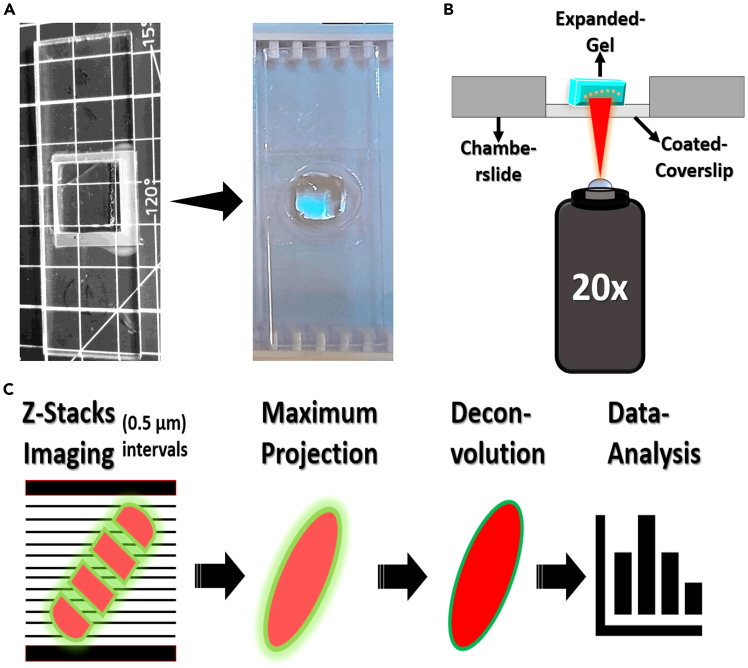
Gel imaging and image processing (A) Imaging chambers containing the expanded gels were stored and transported in a microscope slide box to maintain darkness and minimize photobleaching. (B) Imaging was performed using an inverted confocal laser scanning microscope equipped with a 20×/0.75 NA air objective. Z-stacks were acquired at 0.5 μm intervals using a 16-bit, 512 × 512-pixel frame size at 9× digital zoom. Fluorescence excitation was set at 561 nm for the red channel and 488 nm for the green channel. (C) Confocal Z-stack images were deconvolved using Huygens software, and the resulting image stacks were imported into Fiji. Maximum intensity projections were generated for subsequent pre-processing and quantitative analysis.
11. Expanded Gel/NMJ Imaging:
  a. Set up the Nikon A1/A1R (or equivalent) confocal laser scanning microscope with a 20x/0.75 NA (air objective). Configure the imaging settings to acquire 16-bit images at 512 x 512-pixel resolution. Select Z-stack acquisition with a step size of 0.5 μm. imaging details can be summarized in (Figures 4B and 4C).
    Pixel size was variable across samples, reflecting adjustments in digital zoom to match the field of view to NMJ size.
    ***Note:*** The imaging parameters described above were selected to balance resolution and field of view for whole-mount NMJ visualization and may not strictly conform to Nyquist sampling. Users who wish to maximize spatial resolution are advised to adjust pixel size and Z-step spacing according to the Nyquist criterion.
  b. Use the following laser excitation settings:
     -Alexa Fluor 488 (Green Channel).
     -Alexa Fluor 594 (Red Channel).
  c. Begin by scanning the sample at low magnification (i.e., 4x or 10x objectives) to locate NMJs that are well-labeled and oriented en face (the postsynaptic endplate is visible perpendicular to the imaging axis and reveals the classic pretzel-like morphology).
  d. After selecting the suitable NMJ, choose the higher magnification objective (20x) for imaging.
    ***Note:*** Optimize fluorescence signal by selecting NMJs located close to the bottom surface of the expanded gel, ideally within 40–60 μm of the coverslip. This helps minimize light scattering and signal attenuation caused by increased imaging depth within the gel matrix.
    **CRITICAL:** Expanded gels should remain fully hydrated and be imaged immediately after mounting, as partial dehydration may cause shrinkage and alter the expansion factor. In our workflow, each gel requires approximately 45 min of imaging to capture NMJs within the appropriate focal plane and working distance. Typically, around six gels are processed in one experiment, with each gel maintained in distilled water until the time of imaging.

### Image analysis and quantification

**Timing: Variable**

This section describes the image processing and analysis of expanded NMJ. Deconvolution improves image clarity and resolution, while subsequent morphometric and fluorescence-intensity analyses in Fiji and GraphPad Prism yield accurate structural measurements (after considering the expansion factor).

12. Deconvolution:
  a. Upload confocal Z-stack images into the Huygens software platform.
  b. Apply the Classic Maximum Likelihood Estimation (CMLE) deconvolution algorithm, which is automatically computed based on the microscope’s Point Spread Function (PSF).
13. Image and Data Analysis:
  a. Deconvolved z-stack images need to be loaded into Fiji software and collapsed into maximum intensity projections for pre-processing and measurements as per the following:
  b. AChR measurements^1^ (optional):
    i. Open two TIF files of the same image: one for processing and the other for reference to verify the integrity of thresholding.
    ii. Split the image channels and identify the channel displaying AChRs (typically appearing in cyan in our example) and forming strip- or band-like clusters.
    iii. Zoom in on the region of interest (ROI), then navigate to Image → Adjust → Threshold. Keep the default thresholding mode and manually adjust the sliders to achieve a suitable visual representation, comparing it with the original image for accuracy.
    iv. Go to Process → Binary → Make Binary to convert the thresholded image into binary format.
    v. Use the line measurement tool to measure the widest diameter (“Length”) of each clearly defined AChR strip. Click Measure and record the values shown under the “Length” column in the Results window. Enter these measurements into an Excel spreadsheet under AChR Strips Width (μm).
    vi. On the same image, without altering the zoom or threshold settings, navigate to Process → Binary → Skeletonize.
    vii. Use the line measurement tool to measure the narrowest space between two adjacent, clearly distinguishable AChR strips. Record these values in the Excel spreadsheet under Distance between AChR Strips (μm).
    viii. Still on the skeletonized image, measure the length of individual AChR stripes. Priorities continuous “branches”, and confirm their identity by comparing with the original unprocessed image. Record the measurements under AChR Stripes Length (μm).
    ix. After completing the measurements for one ROI, close the image. Reopen the same image, select a new ROI, and repeat the above steps to collect additional data.
  c. Profile intensity analysis to quantify overlap between AChRs and SV2 or NaV1.4 signals^1^ (optional):
    i. Open a TIF file image of the ExM-NMJ.
    ii. Split the image channels and identify the channel displaying AChRs (typically appearing in cyan or green in our example) and forming strip- or band-like clusters.
    iii. Identify the second image, either NaV1.4 or SV2 (typically appearing in magenta in our example).
    iv. **NOTE:** Fluorescent intensity profiles in our protocol are based on analyzing the signals across the entire NMJ, excluding signal-void regions, to assess the spatial distribution of AChRs, SV2, and NaV1.4. This method provides a more accurate representation than conventional random line analyses.
    v. For both channels, zoom in on the region of interest (ROI), then navigate to Analyze → Plot Profile → Data →Save Data. NOTE: This should be done for each channel separately.
    vi. Enter these measurements into an Excel spreadsheet under AChRs and SV2 or NaV1.4. Note: The number of cells in the two adjacent columns must be the same. Thus, the same ROI should be analyzed exactly despite the chosen channel.
    vii. GraphPad Prism Software (Version 10) was used for all statistical analyses and for preparing graphs in our study.^1^ Instances for those tests, unpaired t-tests were applied for comparisons between groups (AChR Strips Length in Human vs. Mouse). Relationships between fluorescent intensity profile (AChRs signal vs. NaV1.4 signal) were assessed using correlation analysis, reporting Pearson’s r values. An example of this correlation analysis is presented in our previously published Cell Reports Methods paper (A. Ramadan et al., 2025, Figures 6 and 7).^1^

***Note:*** For each NMJ, the same ROI dimensions were applied across both channels to allow direct comparison of signals. However, because NMJ morphology and size vary considerably between samples, ROI dimensions were adjusted between different NMJs to encompass the full structure of interest in each case.

**CRITICAL:** The expansion factor is calculated by dividing the post-expansion gel dimension by the corresponding pre-expansion value. This factor is critical for determining the true size of biological structures and for confirming consistent expansion across samples.

***Note:*** ImageJ measurements are taken on the expanded images, which reflect the inflated dimensions of the gel. To correct for this, we applied the expansion factor (3.4–4.0 times, derived from pre- and post-expansion gel dimensions) during downstream analysis in Excel. Specifically, absolute ImageJ values were divided by the corresponding expansion factor to calculate the true (physical) size of biological structures and to confirm consistency across samples.

## Expected outcomes

**Figure 6:**
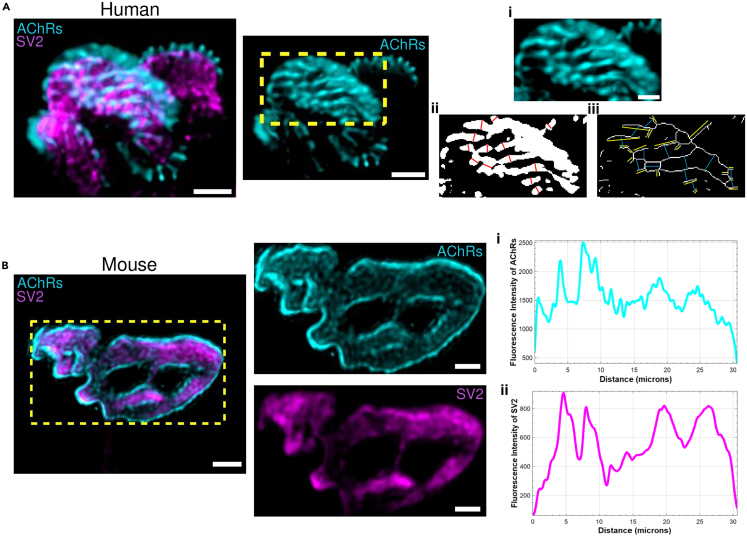
Data analysis methods of expanded NMJs (A) Example of an expanded human NMJ stained with α-Bungarotoxin to label AChRs (cyan) and immunolabeled for SV2 to visualize presynaptic vesicles (magenta). Scale bar= 4 μm. A zoomed-in region (i) illustrates the selected area for measurement. AChR width was quantified on thresholded images (ii), indicated by red bars. AChR strip length and inter-strip spacing were measured on skeletonized images (iii), with yellow bars showing strip length and blue bars indicating spacing. Scale bar in (i)= 2 μm. (B) Mouse NMJ labeled as in (A). The yellow-dotted rectangle denotes the region used for fluorescence intensity profile analysis of AChRs and SV2. Intensity profiles were generated separately for each channel. Scale bar= 4 μm. Scale bar corresponds to the physical size of the expanded sample (not adjusted to expansion factor).

Following the application of this protocol, conventional confocal microscopy is rendered capable of capturing morphological details of the NMJ at the nanoscale. Among the features that exhibit super-resolution characteristics of NMJs are the spatial distribution patterns of AChRs, which are organized into parallel strip-like arrangements, while SV2 signal from synaptic vesicles presents as discrete, punctate hotspots (Figure S2A; Methods videos S1 and S2). Such images underscore the efficacy of the protocol in enhancing the resolving power of standard confocal imaging systems via ExM, thereby enabling the detailed characterization of subcellular structures at a resolution approaching that of traditional super-resolution techniques. Moreover, the acquired morphological features are quantifiable and amenable to statistical analysis, for example, measuring AChR distribution (Figure 6A) and NMJ component overlap or colocalization (Figure 6B). Taken together, this protocol offers a valuable, cost-effective, and accessible means of obtaining super-resolution insights into the NMJ, particularly in contexts where access to advanced imaging modalities are limited.

Methods Video S1. 4x expanded mouse NMJ (AChRs in cyan and Nav1.4 in magenta) captured within a stable gel, related to the troubleshooting section (problem 2, Figure S2.a) The scale bar corresponds to the physical size of the expanded sample (not adjusted based on the expansion factor).

Methods Video S2. 4x expanded mouse NMJ (AChRs in cyan and Nav1.4 in magenta) captured within a drifted gel (downward direction), related to the troubleshooting section (problem 2, Figure S2.b) The scale bar corresponds to the physical size of the expanded sample.

In (Figure S3), we provide a macro- and micro-assessment of NMJ morphology before and after hydrogel expansion, confirming the isotropy and structural fidelity of the process. By re-imaging the same α-BTX–labeled NMJ pre-expansion (after digestion) and post 4x-expansion, both qualitative and quantitative analyses demonstrate uniform enlargement of postsynaptic structures along the X- and Y-axes. The correlation between dimensional changes at the microscopic and macroscopic scales validates the homogeneity of gel behavior, with an average linear expansion of approximately 3.5x. These findings collectively confirm that the ExM protocol preserves architectural integrity and achieves isotropic scaling, thereby supporting the accuracy of all subsequent morphometric analyses.

## Limitations

The principal benefit of ExM lies in its ability to improve spatial resolution through the physical enlargement of the specimen. In order to achieve the full extent of this enhanced resolution it is generally necessary to use high numerical aperture (NA) objectives, as using lower NA lenses constrains the achievable resolving power. However, the 20x air objective was the highest NA objective lens that we could use to image NMJ structures within a reachable focal plane, without needing to force and compress the sample onto the coverslip (nothing that the samples are relatively thick muscle explants from both human and mouse sources). Indeed, mammalian NMJs have a thickness ranging from 20-30 μm (in their unexpanded state) and lie upon muscle fibers with an approximate diameter of ∼80 μm. These dimensions increase significantly following the application of the ExM protocol, since the z-stack becomes deeper during the imaging. Thus, the use of higher magnification/higher NA objectives was not possible due to the physical nature of the samples being imaged.

We acknowledge that re-imaging the exact same NMJ before and after hydrogel expansion is technically challenging and not feasible for every experimental replicate. The ideal scenario for any ExM study is to image the same field of view (FOV) pre- and post-expansion. However, the loss of sample orientation during multiple transfer and washing steps, together with the single-NMJ distribution within each isolated muscle fibre, makes precise FOV alignment virtually impossible. In addition to these spatial and optical challenges, the Z-axis dimension of the sample increases proportionally with the linear expansion factor, often exceeding the working distance of high-numerical-aperture objectives. Consequently, pre- and post-ExM imaging is only realistically achievable for thin or surface-mounted samples where both lateral and axial re-identification remain within the optical range of the imaging system and working distance. Moreover, fluorescence signal deterioration (arising from photobleaching, fluorophore dilution, and digestion-induced degradation) further limits the capacity for repeated imaging of the same NMJ. For this reason, the validation presented here focuses on a representative NMJ imaged post-digestion and post-expansion, demonstrating isotropic scaling and minimal distortion under realistic experimental conditions.

***Note:*** If the experimenter needs to re-image the same NMJ within a whole amount muscle, it will be realistic to perform that after digestion (and considered as the pre-expansion status) Vs post (4x)- expansion.

## Troubleshooting

### Problem 1

Prolonged incubation of samples in the digestion solution may result in the degradation of fluorophores, consequently leading to diminished fluorescence intensity during imaging. Nonetheless, thorough digestion of muscle fibers is essential, as insufficient digestion time can cause visible distortions in the gel during expansion, including the formation of cracks. Refer to the corresponding step in the main protocol **(Steps 7 and 8)** where this issue is most likely to occur.

### Potential solution

In the digestion step, a balance between prolonged and shortened times must be achieved. It is also critical to ensure that the microdissection should yield a relatively small muscle sample (≈10 fibers). Larger muscle samples, as commonly used in standard NMJ staining protocols, are prone to incomplete digestion, resulting in a reduced expansion factor and visible gel distortions, such as cracks.

### Problem 2

Gel drift during imaging due to unstable hydration. Since gels can lose or absorb water during imaging, nanoscale details within the NMJ (Figure S2A) were only achievable when the gel remained stable throughout the imaging process. In contrast, gel drift resulted in a loss of image clarity, as illustrated in (Figure S2B). Therefore, morphological analyses should not be conducted on images of expanded NMJs obtained from drifted gels, as the structural integrity and spatial accuracy of the data may be compromised (even if that will reduce sample numbers). Refer to the corresponding step in the main protocol **(Step 11)** where this issue is most likely to occur.

### Potential solution

To minimize drift, the coverslip floor of the imaging chamber should be coated with 200–300 μl of poly-L-lysine for 1 h before transferring the gel. During imaging, hydration status should be judged by observing the behavior of the gel: If the gel appears to shrink (edges pulling inward) and drift increases, this indicates under-hydration. Correct by adding small water droplets until stability is restored.

If the gel swells or bulges and moves excessively within the chamber, this indicates over-hydration. Correct by gently removing excess liquid with filter paper until stable.

Thus, the choice of corrective action is guided by whether the gel is shrinking or swelling during imaging.

### Problem 3

Incomplete gel polymerization may result in fragile or poorly formed gels that tear easily during handling or fail to retain embedded biological structures. This issue often arises from premature polymerization in the tube (leading to clumps), from delays in applying the gel mixture to the chamber, or from insufficient mixing of polymerization catalysts (APS and TEMED). In such cases, the gel structure becomes mechanically weak or uneven, compromising both expansion and image quality. Refer to the corresponding step in the main protocol **(Step 10)** where this issue is most likely to occur.

### Potential solution

Always prepare the gelation mixture immediately before use by adding APS and TEMED last, mixing quickly but gently to avoid introducing bubbles. Keep the mixture on ice to delay polymerization and minimize thermal variation. Apply the gel solution to the chamber and seal it swiftly to maintain a consistent polymerization environment. If clumps or uneven gel consistency are observed, discard the mixture and prepare a fresh gelation solution. Ensure all components (monomer, APS, TEMED) are within shelf life and stored properly.

### Problem 4

Limited resolution when imaging thick expanded muscle samples due to the restricted working distance of high NA objectives on inverted microscopes. Refer to the corresponding step in the main protocol **(Step 11)** where this issue is most likely to occur.

### Potential solution

If available, an upright confocal microscope equipped with long-working-distance water-dipping objectives can overcome this constraint. For example, a Nikon 60× water-dipping objective (NA 1.0; working distance 2.8 mm) allows imaging of expanded NMJs in aqueous conditions while achieving higher resolution than a 20× air objective.

## Resource availability

### Lead contact

Further information and requests for resources or reagents should be directed to the lead contact, Professor Thomas H. Gillingwater (t.gillingwater@ed.ac.uk), who will respond to reasonable requests.

### Technical contact

This protocol does not have a separate technical contact. All technical queries should be directed to the lead contact, Professor Thomas H. Gillingwater (t.gillingwater@ed.ac.uk).

### Materials availability

This study did not generate new unique reagents.

### Data and code availability

• The datasets generated and analyzed during this study are available from the lead contact or corresponding author upon reasonable request.
• This study does not include any newly developed software or code.
• Further details required to replicate or reanalyze the findings presented in this paper can be obtained from the lead contact or corresponding author upon request.

## Acknowledgments

This work was supported by a Small Project Grant from Anatomy@Edinburgh, University of Edinburgh (awarded to T.H.G. and R.A.J.), and a PhD studentship from King Saud bin Abdulaziz University for Health Sciences, administered via the Saudi Cultural Bureau in London (awarded to A.R. and T.H.G.). Additional support was provided by UK Research and Innovation through a grant awarded to I.J. (MR/S03241X/1). The authors gratefully acknowledge the nursing staff in the operating theaters at St John’s Hospital, Livingston, for their valuable assistance with human tissue collection.

## Author contributions

Conceptualization, A.R., T.M.D.S., I.J., and T.H.G.; formal analysis, A.R.; investigation, A.R.; methodology, A.R., T.M.D.S., A.A. (assisted), and P.A.R.; validation, A.R., T.M.D.S., R.A.J., I.J., and T.H.G.; visualization, A.R.; writing – original draft, A.R.; writing – review and editing, A.R., T.M.D.S., P.A.R., R.A.J., I.J., and T.H.G.; clinical coordination, P.A.R.; ethics coordination, R.A.J.; supervision, T.H.G.

## Declaration of interests

The authors declare no competing interests.
