# Supplementary material for: Protocol for applying expansion microscopy to the study of mammalian neuromuscular junctions
Source: STAR Protoc. 2025 Dec 12;7(1):104272. doi: 10.1016/j.xpro.2025.104272 (PMC12765181; doi:10.1016/j.xpro.2025.104272)
Supplement: Document S1. Figures S1–S3 [file mmc1.pdf]

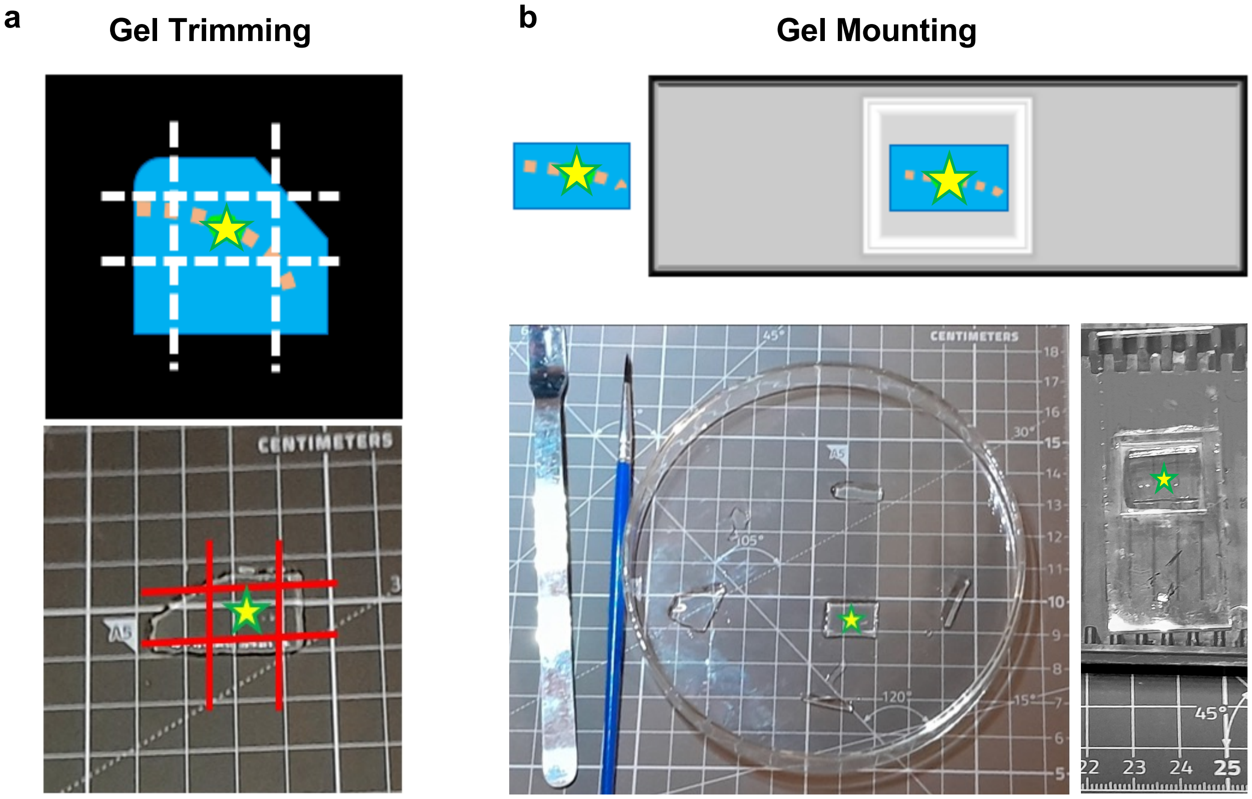

Figure Supplementary 1. Gel trimming and mounting for imaging, related to the gel mounting step (Step 10 and Figure. 4:d).

a. Gel trimming. Schematic **(top)** and photograph **(bottom)** illustrating how non-target regions of the expanded gel are removed to isolate the area containing NMJs. Digested fibres are not visible to the naked eye; trimming is guided by prior knowledge of the NMJ location from fluorophore labelling. Dashed guides indicate trimming lines; the green star marks the NMJ-containing region. **b.** Gel mounting. Schematic **(top)** and photographs **(bottom)** showing placement of the already polymerized and expanded gel segment onto a poly-L-lysine–coated coverslip within the imaging chamber for immediate imaging. The trimmed piece containing the NMJs **(green star)** is centered under a 22 × 22 mm coverslip; the gel is kept hydrated until imaging. Transfer tools (spatula/brush) used for handling are shown.

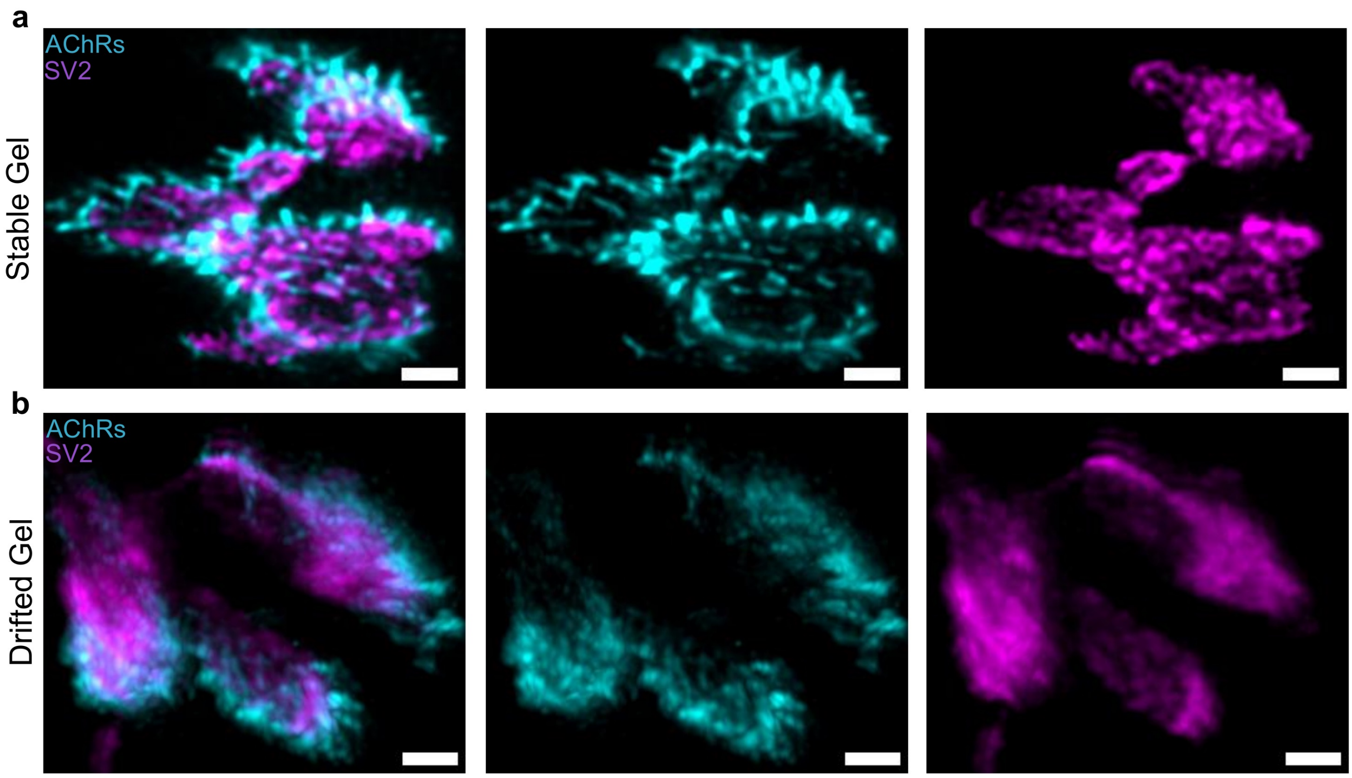

Figure Supplementary 2. Expected outcomes, related to the troubleshooting section (Problem 2).

Representative confocal micrographs showing examples of expanded NMJs (≈4 x) from human muscle biopsies stained with α-Bungarotoxin to reveal AChRs (Cyan), and immunohistochemical labelling of SV2 to reveal presynaptic vesicles (Magenta). **a.** When the gel is stable during imaging, morphological details of NMJs can be captured. **AChRs** appears with the classical striped pattern, while **SV2** signal was distributed as punctuated hotspots. **b.** NMJs in a drifted gel lose these morphological details. Images in **(a)** and **(b)** were acquired with a 20x objective and processed with deconvolution according to the current protocol. The scale bars (4 µm) correspond to pre-expansion (physical) dimensions.

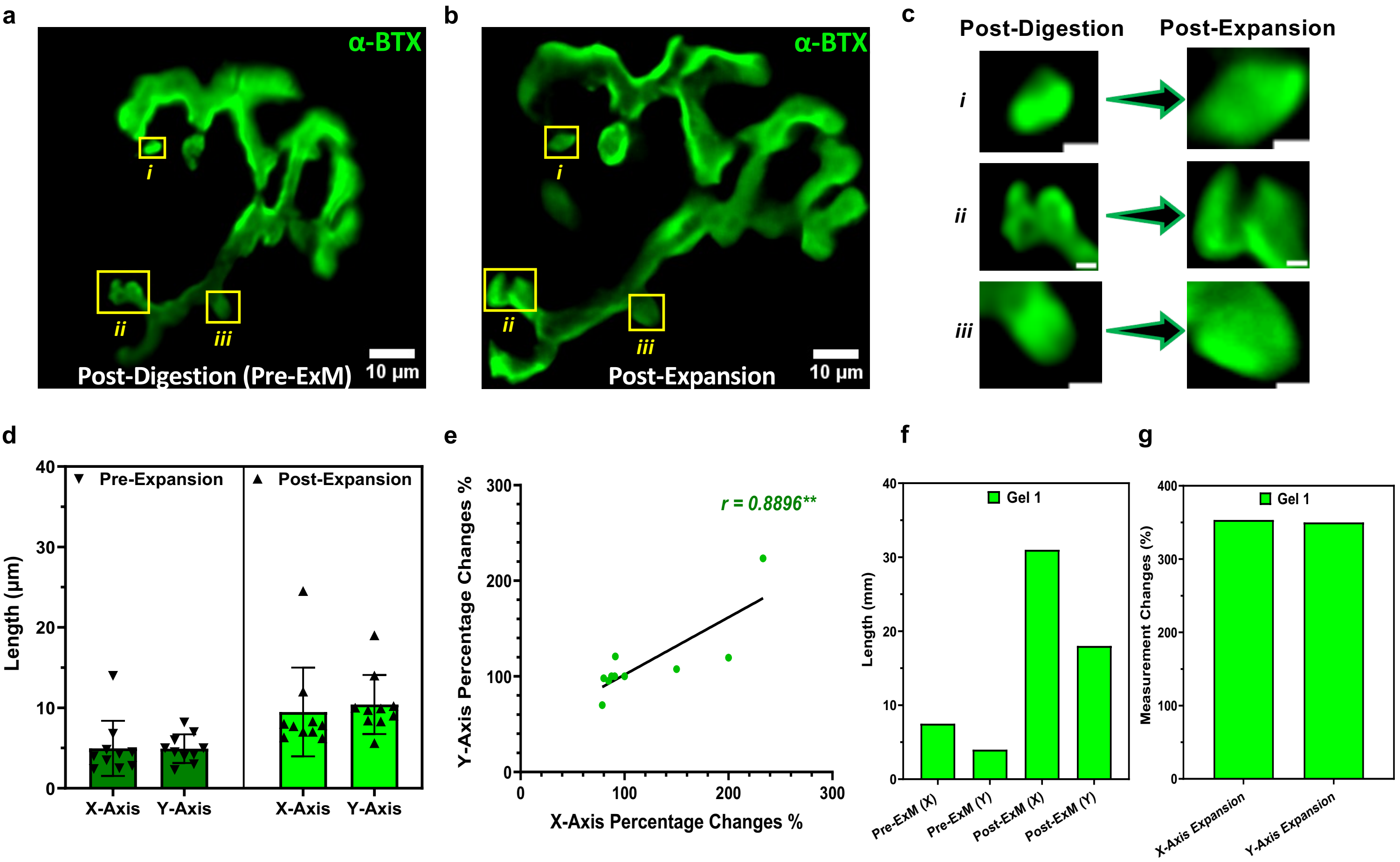

Figure Supplementary 3. Macro and micro-assessment of the expanded NMJ from the mouse dorsal interosseous muscle related to the expected outcomes section.

a. Representative confocal micrograph of a mouse NMJ labelled with α-bungarotoxin (**α-BTX; green**) acquired post-digestion (Pre-ExM). **b.** The same NMJ after post-expansion (Post-ExM) imaging. Both images were re-acquired following the expansion process and subsequently de-noised to compensate for photobleaching due to repeated imaging of the same NMJ. Scale bars, 10 µm. **c.** Higher-magnification examples of selected NMJ subregions **(i–iii)** illustrating morphological differences between post-digestion (pre-4x expansion) and post-expansion states. Structural preservation and proportional enlargement of α-BTX-labelled receptor fields are evident. Scale bars, 2 µm. Note: no apparent tearing or anisotropic distortion is visible. **d.** Quantitative comparison of the absolute linear bouton dimensions along the X- and Y-axis measured in post-digestion **(dark green)** and post-expansion **(bright green)** images. Bars represent mean ± SD, and each data point corresponds to an individual linear measurement (n = 10). No statistically significant differences were observed between X and Y axes, suggesting isotropic expansion. e. Spearman correlation analysis between percentage increases in X- and Y-axis dimensions showed a strong positive correlation *($r = 0.8896$; $p < 0.01$)*, confirming even (isotropic) expansion of the hydrogel at the microscopic level. Each data point represents the corresponding percentage increase derived from the same measurements shown in **(d)**. f. Macroscopic hydrogel measurements of the same sample before (Pre-ExM) and after (Post-ExM) expansion along both axes, demonstrating proportional increases in gel size. **g.** Quantification of expansion percentages in the X- and Y-axis of the same gel, showing an approximate 300-350% linear increase along both axes, consistent with ~3x linear expansion (~3.5x isotropic expansion factor).
